# Real-World Effectiveness and Safety of Tapinarof 1% Cream in Psoriasis: An Observational Study From Bangladesh

**DOI:** 10.7759/cureus.103183

**Published:** 2026-02-07

**Authors:** Samiul Huq, Towhida Noor, Abu Hena Chowdhury, Md. Abdul Latif Khan, Anzirun Nahar Asma, Snigdha Malabeka, Nur A Tasmin Tahnin, Tahmidur Rahman Mahin, Shameem Al Mamun, Afroza Jesmin

**Affiliations:** 1 Department of Dermatology, Psoriasis Awareness Club, Dhaka, BGD; 2 Department of Dermatology and Venereology, Bangladesh Medical University, Dhaka, BGD; 3 Department of Dermatology and Venereology, Combined Military Hospital, Dhaka, BGD; 4 Department of Dermatology and Venereology, Popular Medical College, Dhaka, BGD; 5 Department of Dermatology and Venereology, Sir Salimullah Medical College & Mitford Hospital, Dhaka, BGD; 6 Department of Dermatology, Bangladesh Institute of Dermatology, STD, and AIDS, Dhaka, BGD; 7 Department of Dermatology and Venereology, Sylhet MAG Osmani Medical College, Sylhet, BGD; 8 Department of Dermatology and Venereology, Upazila Health Complex, Dohar, Dhaka, BGD

**Keywords:** bangladesh, combination therapy, psoriasis, real-world study, tapinarof 1% cream

## Abstract

Background

Tapinarof 1% cream, a non-steroidal topical aryl hydrocarbon receptor (AhR) agonist, has demonstrated effectiveness in the treatment of psoriasis. However, real-world data from low-resource settings is scarce. This study evaluated the real-world effectiveness and safety of tapinarof 1% cream in adults with mild to moderate plaque psoriasis, and it explored differences in outcomes between tapinarof monotherapy and tapinarof combined with a moderately potent topical corticosteroid (clobetasone butyrate). Between-group comparisons were prespecified as exploratory and associative due to non-random allocation.

Methods

In this prospective observational study, 122 patients with confirmed plaque psoriasis received tapinarof monotherapy (n = 72) or combination therapy with topical corticosteroids (n = 50). Disease severity was assessed using the Psoriasis Area and Severity Index (PASI) and Physician Global Assessment (PGA) at baseline and at weeks 4, 8, and 12. PASI75 and PASI90 responses were analyzed using odds ratios (ORs) and 95% confidence intervals (CIs). Multivariable-adjusted and longitudinal sensitivity analyses were performed. Adverse events (AEs) were recorded.

Results

At week 12, mean PASI decreased from 6.02 ± 2.40 to 2.78 ± 1.28 in the monotherapy group (p = 0.045) and from 6.46 ± 2.06 to 1.38 ± 1.14 in the combination group (p < 0.001), with a significant between-group difference (p < 0.001). PASI75 was achieved by 30 (60.0%) patients in the combination group and 10 (13.9%) in the monotherapy group (OR = 6.25, 95% CI: 2.38-16.67; p < 0.001). PASI90 responses occurred in 14 (28.0%) and six (8.3%) patients, respectively (OR = 4.35, 95% CI: 1.52-12.50; p = 0.043). Mean changes in PGA differed between groups (p = 0.038). AEs were mild and similar across groups.

Conclusion

Combination therapy was associated with greater clinical improvement than tapinarof monotherapy in this observational cohort; however, these findings represent associations rather than causal effects, and the incremental contribution of tapinarof cannot be isolated because the combination arm included topical corticosteroids. Randomized studies are required to confirm comparative effectiveness.

## Introduction

Psoriasis is a chronic, immune-mediated inflammatory skin disease characterized by erythematous, scaly plaques that can significantly impair a patient’s quality of life. Globally, psoriasis affects approximately 2-3% of the population, with prevalence varying by geographical location and ethnic background [1]. In Bangladesh, psoriasis affects an estimated 0.7% of the population, with plaque-type psoriasis being the most common clinical presentation, accounting for approximately 81% of diagnosed cases [2].

Beyond its cutaneous manifestations, psoriasis imposes a substantial psychosocial burden. The Bangla-validated Psoriasis Disability Index (PDI) has demonstrated strong correlations (r = 0.81) between disease severity and impaired quality of life, emphasizing the condition’s substantial impact on patients' well-being [3]. Hospital-based cross-sectional studies have further established robust associations between higher Psoriasis Area and Severity Index (PASI) scores and increased levels of psychological distress, physical disability, and diminished quality of life among Bangladeshi patients [4]. While nationwide epidemiological data remain limited, institutional studies from urban centers, such as Tairunnessa Medical College Hospital, have documented a prevalence of 1.13% for psoriasis among dermatology patients. This finding underscores the growing clinical and public health concern regarding psoriasis in Bangladesh [2,5,6].

While conventional treatments for psoriasis, including topical corticosteroids, coal tar preparations, systemic immunosuppressants such as methotrexate and cyclosporine, and phototherapy, are often effective, they present significant limitations. These include safety concerns related to long-term use, such as hepatotoxicity, nephrotoxicity, and immunosuppression, as well as the risk of tachyphylaxis and challenges with patient adherence due to complex regimens and cosmetic acceptability issues. These challenges are particularly pronounced in low- and middle-income countries like Bangladesh, where limited access to specialized care, inadequate monitoring resources, and insufficient patient education further complicate disease management and limit the suitability of systemic therapies for long-term use. Such constraints underscore the urgent need for safer, more accessible, and patient-friendly therapeutic options in these settings [7-9]. Consequently, there remains a substantial unmet need for novel, effective, and well-tolerated topical therapies that can provide sustained clinical improvement with minimal adverse effects.

Tapinarof is a novel, non-corticosteroidal topical therapeutic agent recently approved for plaque psoriasis treatment in adults [10]. It functions as an aryl hydrocarbon receptor (AhR) agonist, a mechanism that plays a regulatory role in skin homeostasis and immune modulation. Activation of AhR by tapinarof 1% cream leads to the downregulation of inflammatory cytokines such as IL-17 and IL-23, normalization of skin barrier function, and modulation of keratinocyte proliferation and differentiation [11]. Unlike traditional immunosuppressive agents, tapinarof’s targeted mechanism allows it to exert anti-inflammatory and barrier-restorative effects without the systemic toxicities typically associated with conventional therapies [12].

Several randomized controlled trials (RCTs) conducted in Western populations have demonstrated tapinarof’s efficacy and favorable safety profile in patients with mild to severe plaque psoriasis [13,14]. However, real-world data on its performance in diverse ethnic populations, particularly in South Asia, is limited. Given the differences in genetic predisposition, skin phototype, environmental triggers, and healthcare accessibility, it is crucial to evaluate the clinical effectiveness and tolerability of tapinarof 1% cream in specific populations, such as those in Bangladesh.

The primary objective of this real-world observational study was to assess the change in the PASI over 12 weeks among patients treated with tapinarof 1% cream. Secondary objectives were to assess changes in the Physician Global Assessment (PGA), PASI75 and PASI90 response rates, and treatment-emergent adverse events (AEs). In addition, we exploratorily examined associative differences in outcomes between patients receiving tapinarof monotherapy and those receiving tapinarof in combination with a moderately potent topical corticosteroid (clobetasone butyrate, once daily in the morning, with tapinarof applied once daily at night). Because treatment allocation was based on physician judgment rather than randomization, all between-group comparisons were prespecified as associative and subject to confounding by indication.

## Materials and methods

Study design and setting

This was a six-month, prospective, observational, open-label study conducted at the Psoriasis Awareness Club in Bangladesh from February to September 2024. This specialized dermatology center serves a diverse patient population, consisting of both self-referred individuals and those referred by dermatologists from various regions of the country. One hundred and twenty-two consecutive patients clinically diagnosed with chronic plaque psoriasis were enrolled. Based on individual clinical indications, participants received either tapinarof 1% cream as monotherapy or in combination with topical corticosteroid agents. All patients were followed over a 12-week treatment period, with assessments conducted at four-week intervals.

Study population

The study enrolled patients diagnosed with chronic plaque psoriasis who met specific eligibility criteria. Participants were required to be between 18 and 75 years of age, regardless of sex, with a confirmed clinical and dermoscopic diagnosis of chronic plaque psoriasis. Eligible patients had body surface area (BSA) involvement ranging from 3% to 10% and a PGA score of 2 (mild) or 3 (moderate) at the time of screening. To minimize confounding variables, individuals were included only if they had not received any systemic oral medication for psoriasis within the previous three months and had abstained from topical therapies for at least two weeks before study enrollment. Additionally, all participants were required to provide informed consent and demonstrate their capacity to adhere to study procedures.

**Figure 1:**
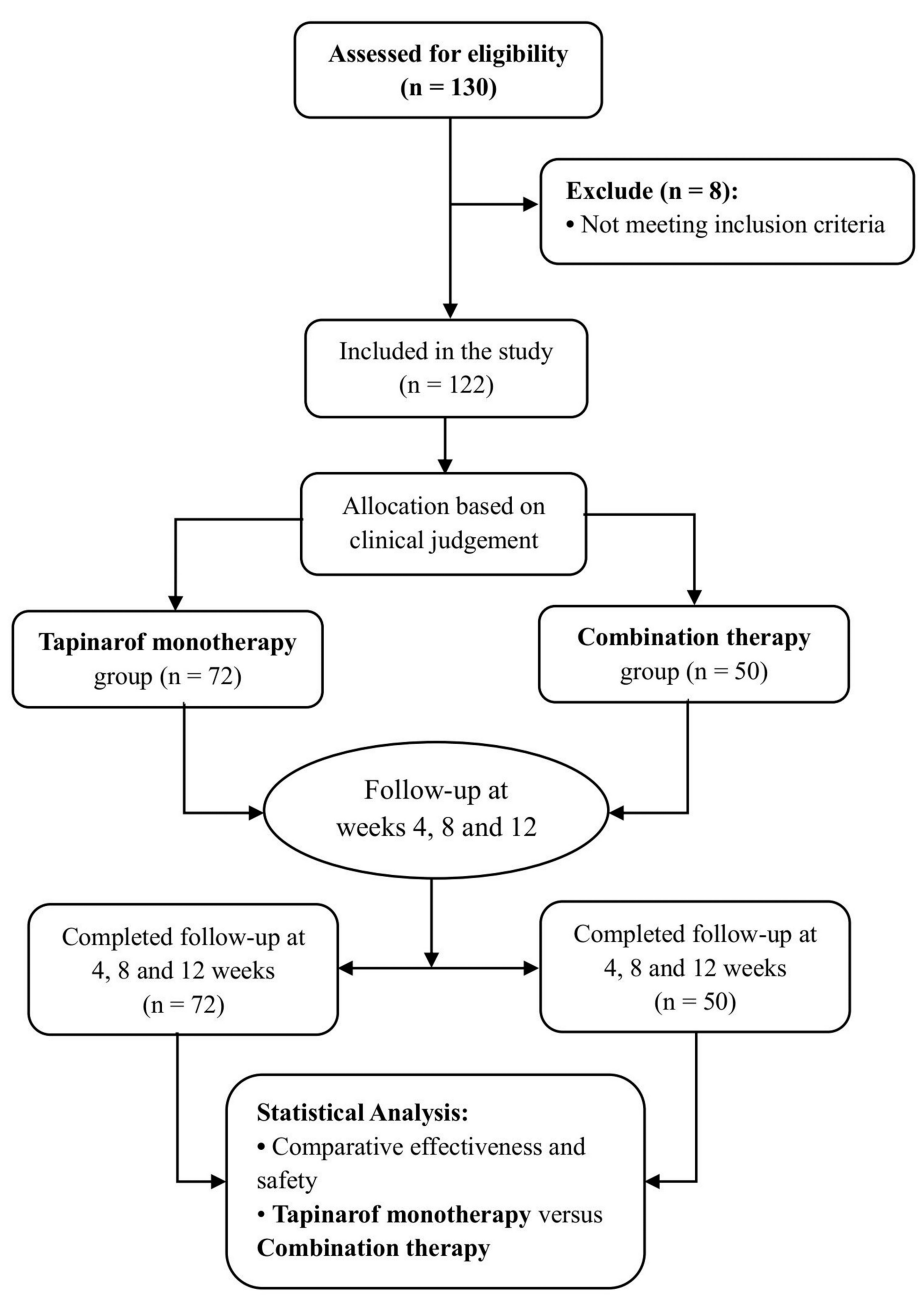
Study flow diagram of patient enrollment, allocation, follow-up, and analysis. The diagram illustrates the participant screening process, the inclusion criteria, and the non-randomized allocation to either tapinarof monotherapy or combination therapy based on clinical judgment. It also includes scheduled follow-ups at weeks 4, 8, and 12, leading to the final comparative efficacy and safety analyses.

Patients with non-plaque variants of psoriasis (e.g., guttate, erythrodermic, or pustular), who were immunocompromised (e.g., with a history of lymphoma, AIDS, or HIV positivity), or who had recent systemic infections requiring treatment within four weeks were excluded. Active skin infections (bacterial, fungal, or viral) occurring within one week before the baseline also warranted exclusion. Additionally, individuals with serious medical or psychiatric conditions that could impede participation, pregnant or lactating women, and those with other skin lesions in the treatment area that might affect assessment or safety were also excluded from the study. A flow diagram illustrating the enrollment, allocation, follow-up, and analysis of study participants is presented in Figure 1.

Disease severity assessment

Psoriasis severity was assessed at baseline and weeks 4, 8, and 12 using the PASI, a validated tool that integrates BSA involvement with lesion characteristics, considering key clinical features such as erythema (E), induration (I), and scaling (S), each scored from 0 (none) to 4 (very severe). These characteristics were applied to four anatomical regions (head, upper limbs, trunk, and lower limbs), weighted by surface area: 10%, 20%, 30%, and 40%, respectively. The PASI was assessed using the following formula [15]:

PASI = [Head: $0.1(E+I+S)\times A$] + [Upper limb: $0.2(E+I+S)\times A$] + [Trunk: $0.3(E+I+S)\times A$] + [Lower limb: $0.3(E+I+S)\times A$]

Here, A denotes the percentage of the surface area involved in each region (0-100%), and the multipliers reflect the percentage of total BSA represented by each region (head: 10%, upper limbs: 20%, trunk: 30%, lower limbs: 40%). The resulting PASI score ranges from 0 to 72, with higher scores indicating more severe disease.

Physician global assessment (PGA)

The PGA was used as a clinical tool to evaluate psoriasis severity and monitor treatment response throughout the study. It employed a static 5-point ordinal scale ranging from 0 (clear), 1 (almost clear), 2 (mild), 3 (moderate), to 4 (severe), based on the degree of erythema, scaling, and plaque elevation [16]. The study showcased patient cases demonstrating treatment results. These results include responses that meet the regulatory endpoint of a PGA score of 0 (clear) or 1 (almost clear), along with at least a 2-point reduction from baseline by week 12.

Bias mitigation

Because this study was open-label, observer bias could have been a limitation. To reduce this risk, the same qualified and trained dermatologist conducted all PASI and PGA assessments at each visit. The evaluator used a standardized scoring protocol, which included high-resolution clinical photographs taken at each visit under consistent lighting and background conditions to support scoring. However, no blinded central review was conducted.

Treatment exposure

All enrolled patients received tapinarof 1% cream, administered either as monotherapy or in combination with a topical corticosteroid.

In the combination therapy group, the treating dermatologist selected the specific topical corticosteroid agent, formulation, and potency according to routine clinical practice and lesion characteristics. The corticosteroids used included clobetasone butyrate 0.05% cream (moderately potent), mometasone furoate 0.1% cream (potent), and betamethasone valerate 0.1% cream (potent). Corticosteroids were applied once daily in the morning to active lesional areas, while tapinarof 1% cream was applied once daily at night. The quantity of corticosteroid applied followed the fingertip unit (around 0.5 g) method. Corticosteroid therapy was prescribed continuously during the 12-week study period, with dose reduction or intermittent use permitted at the discretion of the treating physician in cases of marked clinical improvement (Table 1). 

**Table 1:** Topical corticosteroids used in the combination therapy group ^a^Potency classification according to standard topical corticosteroid potency categories.

Corticosteroid agent	Potency class^a^	Formulation	Application frequency	Application site	Planned duration	Tapering/intermittent use
Clobetasone butyrate 0.05%	Moderate	Cream	Once daily (morning)	Active plaque lesions	Up to 12 weeks	Allowed at physician's discretion
Mometasone furoate 0.1%	Potent	Cream	Once daily (morning)	Active plaque lesions	Up to 12 weeks	Allowed at physician's discretion
Betamethasone valerate 0.1%	Potent	Cream	Once daily (morning)	Active plaque lesions	Up to 12 weeks	Allowed at physician's discretion

Data sources and measurements

All data were collected through face-to-face interviews and clinical assessments conducted by trained staff. Demographic and clinical information (age, sex, smoking history, family history, disease onset, and duration of psoriasis) was obtained from patient interviews and verified with medical records. Dermatologists performed standardized evaluations of psoriasis severity using PASI and PGA, and lesion types (e.g., plaque palmoplantar sebopsoriasis) were documented with photographic support. AEs were monitored at each follow-up, graded according to the Common Terminology Criteria for Adverse Events (CTCAE) version 5.0 [17], and assessed for relatedness. Treatment adherence was assessed qualitatively using patient self-reports and review of treatment diaries at each follow-up visit, applying the same procedure in both study groups. Quantitative adherence metrics were not systematically captured.

Outcome measures

Primary Outcome

The primary outcome measures were to assess changes in PASI scores over 12 weeks of tapinarof 1% cream therapy, either as monotherapy or in combination with topical agents. Additionally, we aimed to compare the proportion of patients achieving PASI75 (defined as a ≥75% improvement in PASI score from baseline) at the final follow-up visit between the two groups.

Secondary Outcomes

Secondary outcome measures included the percentage reduction in PASI score from baseline, calculated for each time point (at baseline, weeks 4, 8, and 12), to evaluate treatment response and changes in PGA at week 12. Additionally, AEs were assessed at each follow-up visit.

Sample size and power calculation

This was an observational study without formal randomization; therefore, a prior sample size calculation based on the expected effect size was not conducted. Instead, 122 eligible and consenting patients were included over a defined six-month period for both tapinarof 1% cream monotherapy and combination therapy, with 72 and 50 patients, respectively. After data collection, a post-hoc power analysis was performed to assess whether the sample size was adequate for the primary outcome - the proportion of patients achieving PASI75 at 12 weeks. Based on the observed group proportions, the calculated statistical power exceeded 90% at α = 0.05, indicating adequate power to detect a between-group difference.

Statistical analysis

No missing data were identified. All primary efficacy and safety analyses were conducted using a per-protocol (PP) approach, which included only participants who completed the full 12-week treatment period and attended all scheduled assessments. This approach was chosen to evaluate treatment effectiveness under optimal adherence conditions in a real-world clinical setting.

Treatment allocation to tapinarof monotherapy or combination therapy was determined by the physician's clinical judgment rather than randomization. As a result, baseline differences in disease chronicity, severity, or physician treatment preference may have influenced group assignment (confounding by indication). Accordingly, all comparative analyses were interpreted as associative rather than causal. Between-group comparisons were performed to explore real-world treatment patterns and outcomes, acknowledging the potential influence of unmeasured confounders.

The distribution of continuous variables was assessed using the Shapiro-Wilk test, along with visual inspection of histograms and Q-Q plots. Categorical variables were compared using Pearson’s chi-square test, while continuous variables were analyzed with the independent t-test or Mann-Whitney U test, as appropriate. PASI scores at all time points, as well as age and body mass index, were normally distributed (p > 0.05). Within-group changes in PASI and PGA scores were analyzed with paired t-tests, and between-group comparisons of mean changes were evaluated using independent t-tests. Comparisons of PASI scores between groups at each follow-up visit were conducted with independent t-tests.

Although the PGA is an ordinal scale, it was analyzed as a continuous variable because its distribution approximated normality at baseline and week 12, and mean change scores were of primary interest. Parametric tests were selected because assumptions of normality were met and sample sizes were sufficient for the central limit theorem to apply, a practice commonly accepted in psoriasis outcomes research. As sensitivity analyses to account for the ordinal nature of the PGA, non-parametric and ordinal regression methods were applied. Between-group differences in change in PGA score were assessed using the Mann-Whitney U test. Proportional odds models were fitted for the week 12 PGA category, adjusting for the same covariates as in the primary adjusted analyses. In addition, a responder analysis was performed, defining response as achievement of a PGA score of 0 or 1 at week 12 with a ≥2 point improvement from baseline.

Categorical outcomes, including PASI75 and PASI90 responses, were compared between groups using Pearson’s chi-square test. Odds ratios (ORs) with 95% confidence intervals (CIs) were calculated to estimate relative treatment response. Absolute risk differences and descriptive number needed to treat (NNT) values were calculated from unadjusted response proportions to aid clinical interpretation.

Multivariable regression analyses were conducted to adjust for potential confounding by indication. Logistic regression models were used to estimate adjusted associations for PASI75 and PASI90, and linear regression models were used to evaluate adjusted associations for changes in PASI and PGA scores. All models included treatment group, baseline PASI score, disease duration, psoriasis subtype, age, sex, and body mass index as covariates. Adjusted estimates were interpreted as associative rather than causal. Psoriasis subtypes were combined into a binary variable (plaque psoriasis versus other subtypes) for multivariable analyses due to low category counts and differences in outcome distributions.

To assess the impact of excluding withdrawals, a sensitivity analysis using an intention-to-treat approach that included all 130 enrolled participants was performed. For PASI75 and PASI90, participants without week 12 assessments were conservatively classified as non-responders (non-responder imputation). For continuous outcomes, last observation carried forward (LOCF) was used to impute missing week 12 PASI and PGA values. Results of the sensitivity analyses were compared with those of the primary PP analyses.

AEs were actively assessed at each scheduled visit (weeks 4, 8, and 12) through patient interviews and targeted dermatologic examinations. Patients could report multiple AEs. For tabulation, events were summarized on a per-patient basis (i.e., the number of patients experiencing at least one event of a given type). The treating dermatologist assessed severity and relatedness to the study treatment using CTCAE v5.0 criteria and standard attribution categories (related, possibly related, or unrelated). In the combination therapy group, corticosteroid-related local AEs (including skin atrophy and telangiectasia) were specifically and prospectively assessed at treated sites. All statistical analyses were performed using STATA version 17 (StataCorp, College Station, TX, USA).

## Results

Of the 130 patients enrolled, 122 (96.7%) completed the full 12-week follow-up. Eight participants withdrew before week 12 due to personal reasons unrelated to treatment. These participants were excluded from the PP analysis. Thus, 72 patients in Group 1 (tapinarof monotherapy) and 50 in Group 2 (combination therapy) were included in the final analysis. No patients discontinued treatment due to AEs (Figure 1). In an intention-to-treat-like sensitivity analysis including all 130 enrolled participants and using conservative non-responder imputation for PASI75 and PASI90 and LOCF for continuous outcomes, the direction and statistical significance of the associations between treatment group and clinical outcomes were unchanged compared with the primary PP analyses (Appendix Table 9).

Baseline characteristics of patients

The mean age of patients in Group 1 (tapinarof monotherapy, n = 72) was 33.92 ± 14.45 years, while in Group 2 (Combination therapy, n = 50), it was 37.12 ± 11.92 years (p = 0.348). The proportion of male patients was 44 (61.11%) in Group 1 and 34 (68.00%) in Group 2 (p = 0.582). The mean body mass index was 24.95 ± 4.72 kg/m² in Group 1 and 25.38 ± 5.69 kg/m² in Group 2, with no statistically significant difference between groups (p = 0.310). A total of eight (11.11%) patients in Group 1 reported a history of smoking, compared to four (8.00%) patients in Group 2 (p = 0.523). A family history of psoriasis was present in 12 (16.67%) patients in Group 1 and 12 (24.00%) in Group 2 (p = 0.479).

Regarding psoriasis subtypes, plaque psoriasis was the most common in both groups: 46 (63.89%) patients in Group 1 and 40 (80.00%) in Group 2. Palmoplantar psoriasis was observed in 18 (25.00%) patients in Group 1 and in six (12.00%) in Group 2. Additionally, four (5.56%) patients in Group 1 had both plaque psoriasis and palmoplantar psoriasis, while three (5.56%) patients in Group 2 had plaque psoriasis along with sebopsoriasis. In Group 1, other types of psoriasis were noted in four patients (5.56%), whereas none were recorded in Group 2. The overall distribution of psoriasis types did not differ significantly between groups (p = 0.117). At baseline, a PGA score of 2 was observed in 36 (50.00%) patients in Group 1 and 30 (60.00%) in Group 2, while a PGA score of 3 was observed in 34 (47.22%) and 18 (36.00%) patients, respectively, with no statistically significant difference between groups (p = 0.678). The median duration of disease in Group 1 was three years (IQR: 2-6), while in Group 2, it was five years (IQR: 4-11), indicating a statistically significant difference (p = 0.039) (Table 2).

**Table 2:** Baseline characteristics of patients with psoriasis receiving tapinarof monotherapy (Group 1) or combination therapy (Group 2) (n = 122) SD: standard deviation; (Q1-Q3): interquartile range; BMI: body mass index; PGA: Physician Global Assessment. Continuous variables are presented as mean ± SD or median (Q1-Q3) and categorical variables as n (%). ^*^Indicates statistical significance at p <0.05.

Variable	Group 1: Tapinarof monotherapy (n = 72)	Group 2: Combination therapy (n = 50)	Test statistic	p-Value
Age (mean ± SD)	33.92 ± 14.45	37.12 ± 11.92	t = 0.91	0.348
Gender (male), n (%)	44 (61.11%)	34 (68.00%)	χ² = 0.30	0.582
BMI (mean ± SD)	24.95 ± 4.72	25.38 ± 5.69	t = 0.29	0.310
Smoking habit, n (%)	8 (11.11%)	4 (8.00%)	NA	0.213
Family history of psoriasis, n (%)	12 (16.67%)	12 (24.00%)	χ² = 0.80	0.479
Psoriasis subtype, n (%)				
Plaque psoriasis	46 (63.89%)	40 (80.00%)	NA	0.117
Palmoplantar	18 (25.00%)	6 (12.00%)		
Plaque and palmoplantar	4 (5.56%)	0 (0.00%)		
Plaque and sebopsoriasis	0 (0.00%)	3 (5.56%)		
Other	4 (5.56%)	0 (0.00%)		
Baseline PGA, n (%)				
PGA = 2	36 (50.00)	30 (60.00)	χ² = 0.78	0.678
PGA = 3	34 (47.22)	18 (36.00)		
Duration of psoriasis, median (Q1-Q3) years	3 (2-6)	5 (4-11)	z = 2.07	0.039^*^

Treatment adherence was monitored through patient self-report and treatment diaries during follow-up visits; however, adherence was not recorded as a quantitative variable and therefore could not be summarized numerically or compared between treatment groups.

Comparison of PASI score changes between treatment groups

At baseline, the mean PASI score was 6.02 ± 2.40 in the monotherapy group and 6.46 ± 2.06 in the combination therapy group. At week 12, PASI scores decreased to 2.78 ± 1.28 and 1.38 ± 1.14, respectively. Within-group analysis showed significant reductions from baseline in the monotherapy group (p < 0.045) and a highly significant decrease in the combination therapy group (p < 0.001) (Table 3).

**Table 3:** Comparison of PASI score changes between tapinarof monotherapy and combination therapy over 12 weeks (n = 122) PASI: Psoriasis Area and Severity Index. Values are presented as mean ± standard deviation (SD). The p-value indicates within-group changes from baseline to week 12. ^*^Indicates statistical significance at p <0.05.

Group	Baseline PASI	PASI at 12 weeks	p-Value
Tapinarof monotherapy (n = 72)	6.02 ± 2.40	2.78 ± 1.28	0.045^*^
Combination therapy (n = 50)	6.46 ± 2.06	1.38 ± 1.14	<0.001^*^

Comparison of PASI scores and percentage reduction over 12 weeks

At baseline, mean PASI scores were 6.02 ± 2.40 in the monotherapy group and 6.46 ± 2.06 in the combination therapy group, indicating no statistically significant difference (p = 0.771). At week 4, the mean scores were 5.26 ± 2.31 for the monotherapy group and 6.08 ± 2.30 in the combination therapy group (p = 0.088). At week 8, the scores were 3.73 ± 1.78 and 3.81 ± 2.28, respectively (p = 0.569). By week 12, PASI scores had decreased to 2.68 ± 1.28 in the monotherapy group and 1.38 ± 1.14 in the combination therapy group, with this difference reaching high statistical significance (p < 0.001). The percentage reduction in PASI scores from baseline to week 12 was 55.76% in the monotherapy group and 77.95% in the combination therapy group, also indicating a highly significant difference between the two groups (p < 0.001) (Table 4).

**Table 4:** Comparison of PASI scores and percentage reduction between tapinarof monotherapy and combination therapy over 12 weeks (n = 122) PASI: Psoriasis Area and Severity Index; SD: standard deviation. Values are presented as mean ± SD or percentage. ^*^Indicates statistical significance at p <0.05.

PASI at various time points	Group 1: Tapinarof monotherapy (n = 72)	Group 2: Combination therapy (n = 50)	p-Value
Baseline (mean ± SD)	6.02 ± 2.40	6.46 ± 2.06	0.771
4 Weeks (mean ± SD)	5.26 ± 2.31	6.08 ± 2.30	0.088
8 Weeks (mean ± SD)	3.73 ± 1.78	3.81 ± 2.28	0.569
12 Weeks (mean ± SD)	2.78 ± 1.28	1.38 ± 1.14	<0.001^*^
% PASI reduction	55.76%	77.95%	<0.001^*^

**Figure 2:**
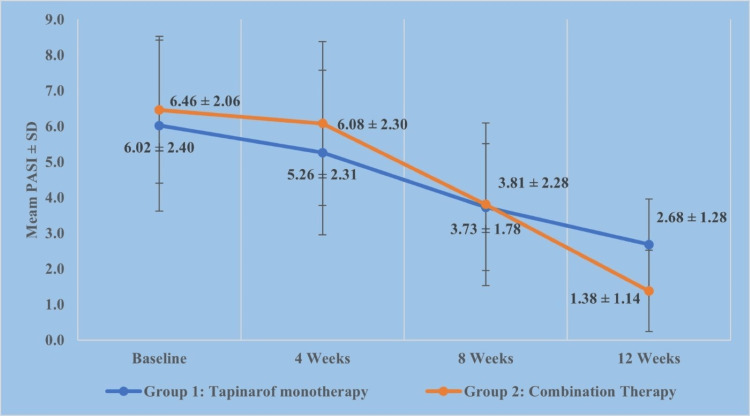
Mean PASI score trajectory over 12 weeks in the tapinarof monotherapy and combination therapy groups. Mean PASI scores are shown at baseline and weeks 4, 8, and 12. Error bars represent standard deviation. A greater reduction in PASI score was observed in the combination therapy group by week 12. PASI: Psoriasis Area and Severity Index.

Mean PASI scores decreased progressively over the 12-week treatment period in both groups, with a greater reduction observed in the combination therapy group by week 12 (Figure 2).

Comparison of PGA score changes between treatment groups

The mean PGA score at baseline was 2.44 ± 0.60 in the monotherapy group and 2.32 ± 0.60 in the combination therapy group. By week 12, scores decreased to 1.63 ± 0.61 and 0.92 ± 0.70, respectively. Within-group analysis showed significant reductions in PGA for monotherapy (p = 0.048) and a highly significant decrease for combination therapy (p < 0.001). A two-sample t-test showed that the mean change was −0.81 ± 0.40 for monotherapy and −1.40 ± 0.76 for combination therapy, with a significant between-group difference (p = 0.038) (Table 5).

**Table 5:** Comparison of PGA score changes between tapinarof monotherapy and combination therapy over 12 weeks (n = 122) PGA: Physician Global Assessment; SD: standard deviation; “-”: not applicable. Values are presented as mean ± SD. ^*^Indicates statistical significance at p <0.05. ^a^ΔPGA represents the mean change from baseline to week 12; ΔPGA = Week 12 PGA − baseline PGA.

Group	Baseline PGA (mean ± SD)	PGA at 12 weeks (mean ± SD)	p-Value (within-group)	Mean change (ΔPGA)^a^ (mean ± SD)	p-Value (between-group)
Tapinarof monotherapy (n = 72)	2.44 ± 0.60	1.63 ± 0.61	0.048^*^	−0.81 ± 0.40	0.038^*^
Combination therapy (n = 50)	2.32 ± 0.60	0.92 ± 0.70	<0.001^*^	−1.40 ± 0.76	-

PASI response and odds ratios

**Figure 3:**
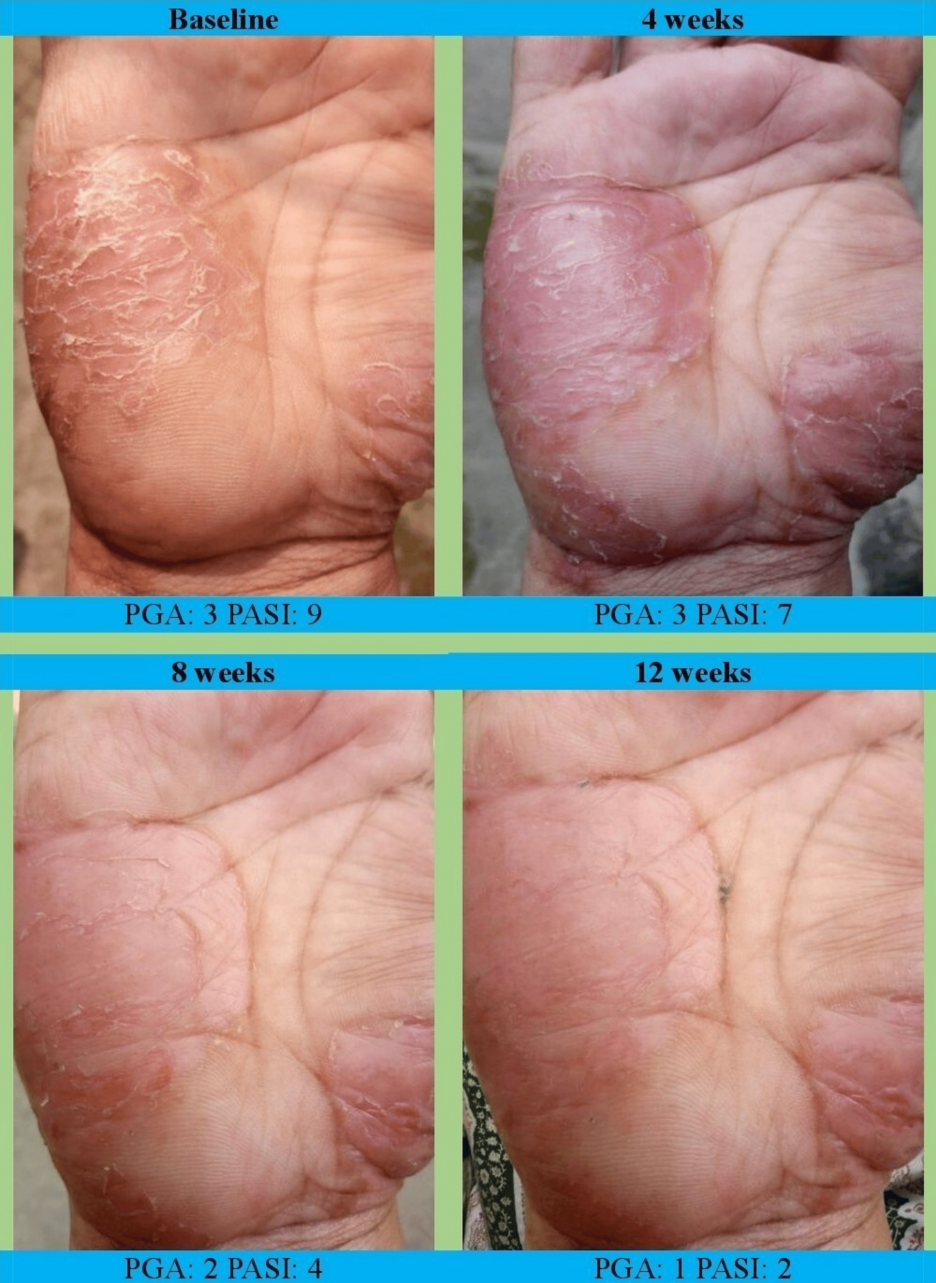
Serial clinical photographs demonstrating treatment response over 12 weeks in a patient with palmoplantar psoriasis treated with tapinarof 1% cream. Representative images show progressive clinical improvement from baseline to weeks 4, 8, and 12, with visible reductions in erythema, scaling, and plaque thickness. The corresponding PGA and PASI scores for each time point indicate sustained improvement throughout the treatment period. PGA: Physician Global Assessment; PASI: Psoriasis Area and Severity Index.

A representative example of clinical improvement over time in a patient with palmoplantar psoriasis treated with tapinarof 1% cream is shown in Figure 3. At week 12, PASI75 response was achieved by 30 (60.00%) patients in the combination therapy group (30/50) and by 10 (13.88%) patients in the tapinarof monotherapy group (10/72). Compared with monotherapy, combination therapy was associated with significantly higher odds of achieving PASI75 (odds ratio [OR] = 6.25; 95% CI: 2.38-16.67; χ² = 14.00, p < 0.001) (Table 6). The absolute risk difference between groups was 46.1%, corresponding to a descriptive NNT of three, calculated from unadjusted proportions.

**Table 6:** PASI75 and PASI90 responses at Week 12 in tapinarof monotherapy and combination therapy, with OR and 95% CI (n = 122) PASI: Psoriasis Area and Severity Index; OR: odds ratios; CI: confidence intervals. PASI75 and PASI90 represent ≥75% and ≥90% improvement from baseline PASI scores, respectively. χ² tests (df = 1) were used for group comparisons. *Indicates statistical significance at p <0.05. ^a^OR >1 represent higher odds of achieving the outcome in the combination therapy group relative to the tapinarof monotherapy group.

Outcome	Tapinarof monotherapy (n = 72); n (%)	Combination therapy (n = 50); n (%)	Odds ratio (95% CI)^a^	χ² (df = 1)	p-Value
PASI75	10 (13.88%)	30 (60.00%)	6.25 (2.38-16.67)	14.00	<0.001^*^
PASI90	6 (8.33%)	14 (28.00%)	4.35 (1.52-12.50)	4.10	0.043^*^

For PASI90, response was observed in 14 (28.00%) patients receiving combination therapy (14/50) and in six (8.33%) patients receiving monotherapy (6/72). Combination therapy was also associated with significantly higher odds of achieving PASI90 compared with monotherapy (OR = 4.35; 95% CI: 1.52-12.50; χ² = 4.10, p = 0.043) (Table 6). The absolute risk difference was 19.7%, corresponding to a descriptive NNT of six. These absolute measures were calculated from unadjusted proportions and should be interpreted cautiously, given the non-randomized observational study design.

Multivariable-adjusted associations between treatment group and clinical outcomes

After adjustment for baseline PASI score, disease duration, psoriasis subtype (collapsed as plaque psoriasis versus other subtypes), age, sex, and body mass index, combination therapy was associated with significantly higher odds of achieving PASI75 at week 12 compared with tapinarof monotherapy (adjusted odds ratio (aOR) = 8.58, 95% CI 1.82-40.48; p = 0.007). Similar results were observed in sensitivity analyses excluding body mass index.

After adjustment for the same covariates, combination therapy was associated with higher odds of achieving PASI90 at week 12; however, this association did not reach statistical significance (aOR = 5.11, 95% CI 0.71-36.69; p = 0.105). In a sensitivity analysis excluding body mass index, the direction and magnitude of the association were similar (aOR = 6.19, 95% CI 0.98-39.04; p = 0.052).

After adjustment for baseline PASI score, disease duration, psoriasis subtype (collapsed as plaque psoriasis versus other subtypes), age, sex, and body mass index, combination therapy was associated with a significantly greater reduction in PASI score compared with monotherapy (adjusted β = 23.86, 95% CI 10.38-37.34; p = 0.001). Similar results were observed in sensitivity analyses excluding body mass index.

After adjustment for baseline PASI score, disease duration, psoriasis subtype (collapsed as plaque psoriasis versus other subtypes), age, sex, and body mass index, the difference in change in PGA score between combination therapy and monotherapy was not statistically significant (adjusted β = 0.36, 95% CI −0.12 to 0.83; p = 0.140). Findings were consistent in sensitivity analyses excluding body mass index (Table 7).

**Table 7:** Multivariable-adjusted associations between treatment group and clinical outcomes All models were adjusted for baseline PASI score, disease duration, psoriasis subtype (collapsed as plaque psoriasis versus other subtypes), age, sex, and body mass index. Adjusted estimates represent associations and do not imply causal effects. CI: confidence interval; PASI: Psoriasis Area and Severity Index; aOR: adjusted odds ratio; PGA: Physician Global Assessment. *Indicates statistical significance at p <0.05.

Outcome	Effect measure	Adjusted effect for combination therapy vs monotherapy (95% CI)	p-Value
PASI75	aOR	8.58 (1.82 to 40.48)	0.007^*^
PASI90	aOR	5.11 (0.71 to 36.69)	0.105
ΔPASI	Adjusted β	23.86 (10.38 to 37.34)	0.001^*^
ΔPGA	Adjusted β	0.36 (−0.12 to 0.83)	0.140

Sensitivity analyses accounting for the ordinal nature of PGA

In a non-parametric comparison of change in PGA score, there was no statistically significant difference between treatment groups (Mann-Whitney U test, p = 0.083). In proportional odds regression for week 12 PGA category adjusted for baseline PASI score, disease duration, psoriasis subtype (plaque versus other subtypes), age, sex, and body mass index, treatment group was not significantly associated with week 12 PGA category (adjusted proportional odds ratio not significant, p = 0.429).

In a responder analysis defining response as achievement of PGA 0 or 1 at week 12 with at least a 2-point improvement from baseline, treatment group was significantly associated with responder status (aOR = 2.23, 95% CI 1.14-4.38; p = 0.019). However, this finding should be interpreted cautiously and in conjunction with the ordinal and non-parametric analyses, which did not demonstrate a statistically significant between-group difference in overall PGA category or change.

Longitudinal mixed-effects analyses of PASI and PGA trajectories

In mixed-effects models including fixed effects for treatment group, time, and a treatment group × time interaction and adjusting for baseline PASI score, disease duration, psoriasis subtype (plaque versus other subtypes), age, sex, and body mass index, a significant treatment group × time interaction was observed for PASI at week 12 (interaction coefficient = 1.57, 95% CI 0.58-2.56; p = 0.002), indicating a greater improvement in PASI over time in the combination therapy group compared with the monotherapy group. The treatment group × time interactions at weeks 4 and 8 were not statistically significant.

In contrast, in the corresponding mixed-effects model for PGA (baseline and week 12 measurements), the treatment group × time interaction was not statistically significant (interaction coefficient = −0.32, 95% CI −0.74 to 0.10; p = 0.131), indicating no evidence of a differential PGA trajectory between the two treatment groups over the study period.

These longitudinal findings were consistent with the primary PP and multivariable adjusted analyses.

Comparison of AEs between groups

At the end of the study, treatment-emergent AEs were reported in eight patients (11.11%) in the monotherapy group and seven patients (14.00%) in the combination therapy group, for a total of 15 patients (12.30%) experiencing AEs. More than one AE could occur in the same patient; however, Table 6 presents the number of patients experiencing each AE category.

Pruritus was reported in three (4.17%) patients receiving monotherapy and three (6.00%) receiving combination therapy. Folliculitis occurred in two (2.78%) patients in the monotherapy group and one (2.00%) patient in the combination therapy group. Erythema was observed in three (4.17%) and two (4.00%) patients, respectively. Hypopigmentation was reported in one (2.00%) patient in the combination therapy group and in none of the monotherapy patients.

All AEs were mild in severity (CTCAE Grade 1). Based on the investigator's assessment, the majority of events were considered related or possibly related to the study treatment. Most events were first noted during the early treatment period (within the first 4-8 weeks of therapy). In the combination therapy group, no cases of corticosteroid-associated skin atrophy or telangiectasia were observed during follow-up.

The single case of hypopigmentation in the combination therapy group occurred at a treated site and was assessed by the investigator as possibly related to topical therapy, potentially exacerbated by concomitant topical corticosteroid exposure (Table 8).

**Table 8:** Comparison of adverse events between tapinarof monotherapy and combination therapy (n = 122) Values are presented as the number of patients experiencing at least one event of the specified type (percentage). Individual patients could experience more than one adverse event. Severity and relatedness were assessed by the investigator according to CTCAE v5.0. In the combination therapy group, corticosteroid-related local adverse effects (including skin atrophy and telangiectasia) were specifically monitored.

Adverse event	Tapinarof monotherapy (n = 72)	Combination therapy (n = 50)	Total (n = 122)
Pruritus, n (%)	3 (4.17%)	3 (6.00%)	6 (4.91%)
Folliculitis, n (%)	2 (2.78%)	1 (2.00%)	3 (2.46%)
Erythema, n (%)	3 (4.17%)	2 (4.00%)	5 (4.10%)
Hypopigmentation, n (%)	0 (0%)	1 (2.00%)	1 (0.82%)
Total adverse events, n (%)	8 (11.11%)	7 (14.00%)	15 (12.30%)

## Discussion

This study evaluated the real-world efficacy and safety of tapinarof 1% cream in patients with chronic plaque psoriasis, comparing outcomes between those receiving tapinarof 1% cream monotherapy and those receiving it in combination with moderate-potency topical corticosteroids. The use of topical corticosteroid agents as part of combination therapy reflects common prescribing practices in Bangladesh for managing recalcitrant or extensive plaque psoriasis. While corticosteroids act via glucocorticoid receptors to reduce inflammation, tapinarof 1% cream targets the AhR pathway, potentially offering complementary mechanisms of action. Although this could contribute to additive effects, the lack of protocolized application timing limits definitive interpretation of synergy [18,19]. 

At baseline demographic and clinical characteristics, patients in both treatment groups were generally comparable. Although the average age was slightly higher in the combination therapy group (37.12 years) than in the monotherapy group (33.92 years), this difference was not statistically significant. Male predominance was observed in both groups, with similar proportions of smokers and individuals with a family history of psoriasis. Plaque psoriasis was the most common subtype in both groups, although palmoplantar psoriasis was more frequent in the monotherapy group. Less common subtypes appeared only in Group 1. Notably, disease duration was significantly longer in the combination therapy group, with a median of five years compared to three years in the monotherapy group. Because treatment allocation was based on physician judgment and the combination group had a longer baseline disease duration, the observed differences between groups may partly reflect confounding by indication rather than treatment-related differences alone.

A notable observation was the delayed yet significant PASI score reduction in the combination therapy group, most evident at week 12 (1.38 ± 1.14), which was highly significant (p < 0.001) and lower than in the monotherapy group (2.78 ± 1.28). At weeks 4 and 8, mean PASI scores between the two groups were similar, with the combination group even showing slightly higher scores than the monotherapy group. This non-linear pattern may suggest a delayed synergistic effect or a cumulative anti-inflammatory response. Prior studies indicate that AhR-target gene modulation by tapinarof 1% cream may enhance corticosteroid-mediated anti-inflammatory effects over time [12,19,20]. Importantly, the greater PASI improvement observed with combination therapy remained consistent after multivariable adjustment, longitudinal mixed-effects modeling, and intention-to-treat-like sensitivity analyses, supporting the robustness of the observed association.

PGA assessments demonstrated a consistent pattern of greater improvement with combination therapy by week 12. PGA scores declined more sharply in the combination group (−1.40 ± 0.76) than in the monotherapy group (−0.81 ± 0.40), with a significant between-group difference (p = 0.038). These parallel improvements in objective lesion severity (PASI) and clinician-rated global appearance (PGA) reinforce the greater overall therapeutic impact of combination therapy over the 12-week period, aligning with previous evidence; improvements in PASI and PGA were consistent over time [21,22]. However, differences in PGA outcomes were attenuated after multivariable adjustment and were not consistently supported by ordinal, non-parametric, or longitudinal sensitivity analyses, suggesting that incremental improvement in PGA may be less robust than that observed for PASI.

In this study, both PASI75 and PASI90 response rates at week 12 were significantly higher in patients receiving combination therapy compared to those treated with tapinarof 1% cream monotherapy (60.00% vs. 13.89%, respectively) (χ² = 14.00, p < 0.001). Similarly, PASI90 responses were attained by 28.0% in the combination group versus 8.3% in the monotherapy group (χ² = 4.10, p = 0.043). The odds of achieving both PASI75 and PASI90 were significantly lower in the monotherapy group, suggesting an enhanced therapeutic effect when tapinarof 1% cream is paired with topical corticosteroids and a higher likelihood of response under real-world conditions, reflecting a deeper level of disease clearance. While tapinarof 1% cream has demonstrated higher efficacy as monotherapy in phase 3 RCTs [12,23], those trials were conducted under tightly controlled conditions with standardized protocols and narrowly selected populations. In contrast, the current real-world observational design reflects pragmatic clinical settings where individualized treatment plans, including combination regimens, are more common. To our knowledge, this is one of the first studies in Bangladesh to investigate tapinarof 1% cream versus combination with a topical corticosteroid to treat patients with chronic plaque psoriasis, suggesting that adjunctive topical agents may be associated with enhanced outcomes in real-world settings in low- and middle-income countries (LMICs), like Bangladesh. Although absolute risk differences and descriptive NNT estimates were presented to aid clinical interpretation, these measures were derived from unadjusted proportions and should be interpreted cautiously in the context of this observational study design.

AEs were infrequent and generally mild across both treatment groups. The most commonly reported events included pruritus, folliculitis, and erythema, with a slightly higher numerical incidence in the tapinarof 1% cream monotherapy group. Importantly, all AEs were classified as Grade 1 in severity according to CTCAE v5.0 and were considered treatment-related but non-serious. No serious AEs or treatment discontinuations occurred, underscoring the favorable safety profile of tapinarof 1% cream when used alone or in combination with other topical agents, as noted by Lebwohl MG et al. [12] and Strober B et al. [14]. The findings of this study are consistent with previous clinical trials demonstrating tapinarof’s efficacy as a topical AhR agonist with a novel mechanism of action and favorable tolerability profile [14]. Importantly, the study adds real-world data from a South Asian population, where evidence on tapinarof 1% cream is currently limited.

Limitations

This study has several limitations. Treatment allocation was not randomized and was based on physician judgment, introducing confounding by indication; therefore, observed between-group differences may reflect baseline disease characteristics, including the significantly longer disease duration in the combination group and potentially more refractory disease, rather than treatment effects alone. Although this design reflects real-world practice, it limits causal inference despite multivariable adjustment and sensitivity analyses.

The combination therapy arm included multiple topical corticosteroid agents of varying potencies, leading to inconsistent exposure and making it difficult to attribute effects to a specific corticosteroid regimen. Furthermore, baseline BSA involvement, prior topical or systemic treatment history, comorbidity profiles, and lesion site distribution were not systematically recorded, preventing their incorporation into adjusted analyses. Finally, adherence was assessed qualitatively via patient self-report and treatment diaries; the absence of quantitative adherence metrics precluded a formal comparison of adherence between treatment groups.

## Conclusions

In this real-world observational study, tapinarof 1% cream was associated with clinically meaningful improvement and a favorable tolerability profile in adults with mild to moderate plaque psoriasis when used as monotherapy and when used in combination with topical corticosteroids. The combination regimen was associated with higher PASI75 and PASI90 response rates and greater improvement by week 12 compared with tapinarof monotherapy.

These findings should be interpreted as associative rather than causal, given the non-randomized treatment allocation, PP primary analyses, and the potential for residual confounding. In addition, because the combination arm included topical corticosteroids - an established and effective therapy for psoriasis - the incremental contribution attributable specifically to tapinarof cannot be isolated in this study. Further adequately powered RCTs in the Bangladeshi population are warranted to confirm these associations and to clarify the added benefit of tapinarof when used alongside topical corticosteroids.

## Appendices

**Table 9:** Intention-to-treat–like sensitivity analysis of treatment effects (n = 130) ITT: intention-to-treat; aOR: adjusted odds ratio; PASI: Psoriasis Area and Severity Index; PGA: Physician Global Assessment; LOCF: last observation carried forward. All models were adjusted for baseline PASI score, disease duration, psoriasis subtype (collapsed as plaque psoriasis versus other subtypes), age, sex, and body mass index. For PASI75 and PASI90, participants without Week 12 assessments were classified as non-responders (non-responder imputation). For continuous outcomes (ΔPASI and ΔPGA), Week 12 values were imputed using the LOCF method. Adjusted estimates represent associations and do not imply causal effects.

Outcome	Effect measure	ITT-like adjusted effect for combination therapy vs monotherapy (95% CI)	p-Value
PASI75 (non-responder imputation)	aOR	7.94 (1.69 to 37.25)	0.009
PASI90 (non-responder imputation)	aOR	4.82 (0.71 to 32.64)	0.112
ΔPASI (LOCF)	Adjusted β	22.31 (9.84 to 34.78)	0.001
ΔPGA (LOCF)	Adjusted β	0.32 (−0.14 to 0.78)	0.171
